# Repair of patellar tendon rupture after total knee arthroplasty using knotless suture bridge technique: a case report

**DOI:** 10.1093/jscr/rjac167

**Published:** 2022-06-09

**Authors:** Toshihiro Seki, Eiichi Shiigi, Kazushige Seki, Koji Yoshida, Tomoya Okazaki, Kazuya Uehara, Hiroshi Tanaka, Takashi Sakai

**Affiliations:** Department of Orthopedic Surgery, Yamaguchi University Graduate School of Medicine, Ube, Japan; Department of Orthopedic Surgery, Yamaguchi Prefectural Grand Medical Center, Hofu, Japan; Department of Orthopedic Surgery, Yamaguchi Prefectural Grand Medical Center, Hofu, Japan; Department of Orthopedic Surgery, Yamaguchi University Graduate School of Medicine, Ube, Japan; Department of Orthopedic Surgery, Yamaguchi Prefectural Grand Medical Center, Hofu, Japan; Department of Orthopedic Surgery, Yamaguchi Prefectural Grand Medical Center, Hofu, Japan; Department of Orthopedic Surgery, Yamaguchi Prefectural Grand Medical Center, Hofu, Japan; Department of Orthopedic Surgery, Yamaguchi Prefectural Grand Medical Center, Hofu, Japan; Department of Orthopedic Surgery, Yamaguchi University Graduate School of Medicine, Ube, Japan

## Abstract

Few studies have reported on the use of knotless suture anchors to treat patellar tendon rupture from tibial tuberosity after total knee arthroplasty (TKA). We report a case of patellar tendon rupture in an 82-year-old female. She fell 8 weeks after bilateral TKA and presented with a patellar tendon rupture. A knotless suture anchor and a fully threaded Twist-In knotless anchor with flat-braided suture were used to repairing the patellar tendon. Complications related to the extension mechanism after TKA can lead to disastrous consequences. This surgical procedure is a safe and good treatment option to repair patellar tendon rupture after TKA.

## INTRODUCTION

Disruption of the extensor mechanism after total knee arthroplasty (TKA) is rare and occurs in 0.17–2.5% of patients [1–3] and is challenging to treat. Extensor mechanism disorders are divided into three types based on the rupture site: quadriceps tendon rupture, patellar fracture and patellar tendon rupture. To the best of our knowledge, this is the first report about the repair of the patellar tendon to the tibial tuberosity for patellar tendon rupture after TKA using fully threaded Twist-In knotless anchors and flat-braided sutures.

## CASE PRESENTATION

**Figure 1:**
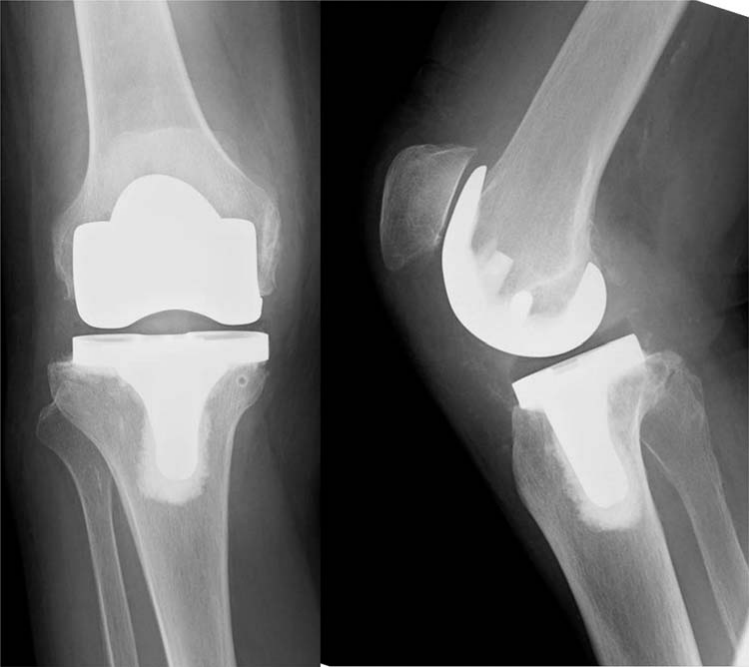
X-ray examination showed a patellar alta.

An 82-year-old woman underwent bilateral TKA for Grade IV knee osteoarthritis. The surgery was performed through a midline gentle curved incision and a trivector approach. Cemented medial pivot implants (Medacta^®^ GMK-Sphere, Medacta International: Castel S. Pietro, Switzerland) were placed. No resurfacing was performed because of the anterior tilt of the patella during surgery. Recovery was uneventful, and the patient was able to walk comfortably without support 3 weeks post-operatively. After 1 week, she felt pain in the right knee joint without any specific triggers. Her gait was stiff, and she needed support to walk. Range of motion testing showed knee extension of −35° and knee flexion of 80°. There was no warmth or redness in the right knee. Tenderness at the tibial tuberosity was mild, and radiographic examination showed patellar alta (Fig. 1).

**Figure 2:**
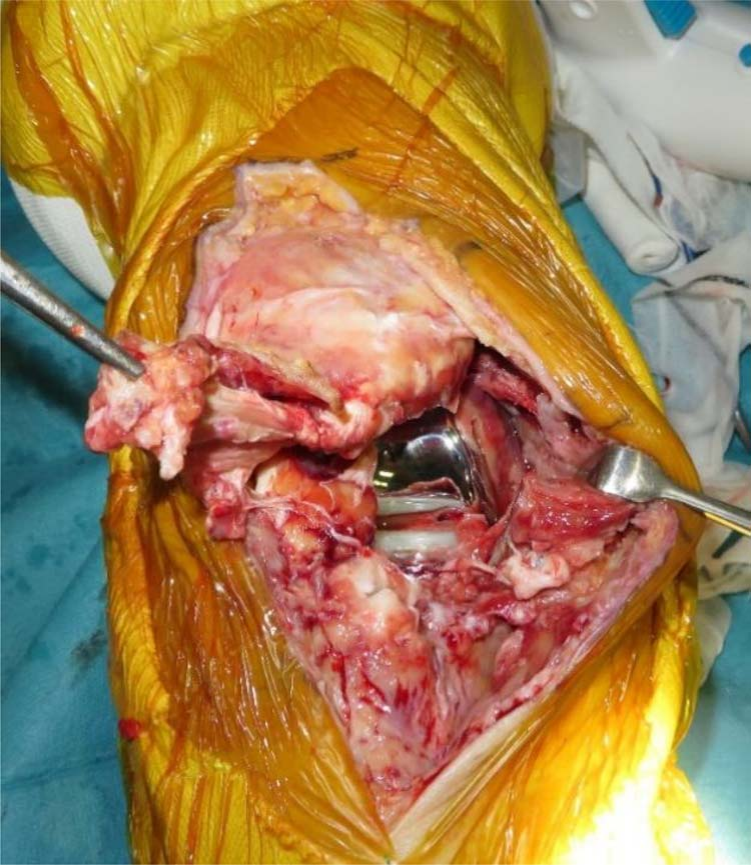
The patellar tendon was found to be ruptured and completely detached from the tibial tuberosity.

**Figure 3:**
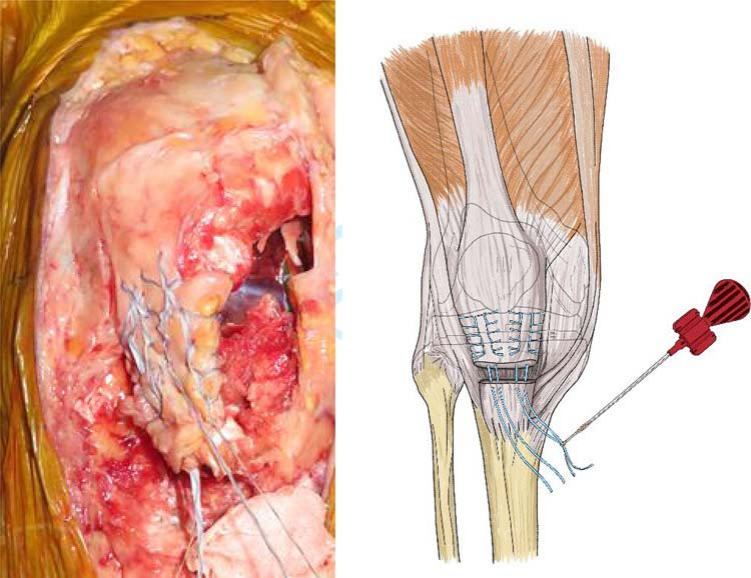
The patellar tendon was completely detached from the tibial tuberosity, and Krakow sutures were applied to both sides of the tendon using flat-braided sutures.

**Figure 4:**
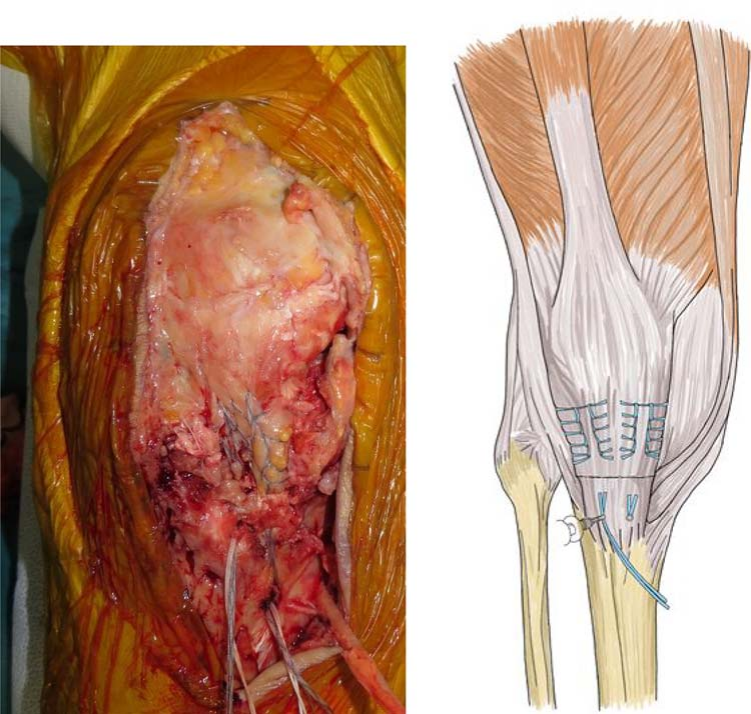
The flat-braided sutures were fixed to the tibial tuberosity using a fully threaded Twist-In knotless anchor (SwieveLock^®^); after pulling the tendon to the tuberosity, it was fixed with two anchors on both sides of the tuberosity.

Patellar tendon avulsion was diagnosed, and the knee was revised through the same incision as for the previous surgery. The patellar tendon was found to be ruptured and completely detached at the tibial tuberosity (Fig. 2). Krakow sutures were applied to both sides of the tendon using flat-braided sutures (Fig. 3). The flat-braided sutures were fixed to the tibial tuberosity using a fully threaded Twist-In knotless anchor (SwieveLock^®^). After pulling the tendon to the tuberosity, it was fixed with two anchors on both sides of the tuberosity (Fig. 4).

**Figure 5:**
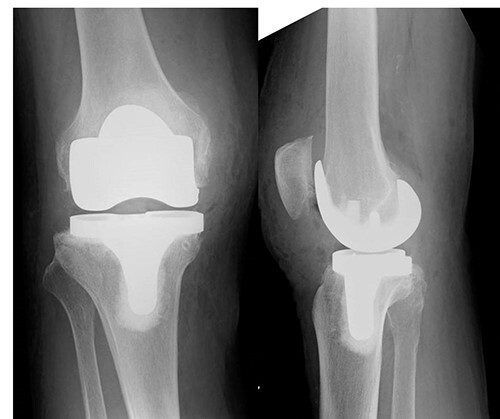
X-ray, 3 months post-operatively, did not show any patellar alta.

After hemostasis, the wound closed in layers. The patient had a cylinder cast for 4 weeks and was allowed full weight-bearing. At 4 weeks post-operatively, the knee was immobilized, and dynamic quadriceps exercises were initiated. The patient regained pain-free motion from 0° to 100° without extension lag. She could walk without a cane 3 months post-operatively. X-rays also showed no patellar alta (Fig. 5).

## DISCUSSION

Disruption of the extensor mechanism leads to loss of the knee function and can occur at any site along with the extensor mechanism; however, the patellar tendon rupture is most likely due to the damage of the patellar ligament insertion after TKA [1–4]. The extent of the disorder of the extensor mechanism, including patella and quadriceps tendon, determines the degree of functional loss due to partial or total tear, whether the disorder is acute or chronic, the availability of viable tissue for direct primary repair or augmentation and the health status of the patient [5, 6].

Surgical treatment includes direct repair only by suturing, stapling or wiring the tendon to the tuberosity and primary repair by reconstruction with biological or synthetic grafts or allografts of the extensor mechanism [1–6]. Direct repair of the disrupted extensor mechanism with sutures, staples or wires has shown encouraging results in patients whose knees have not undergone TKA [3, 6]. Primary repair of patellar tendon ruptures rarely results in recovery of extensor function after TKA [1, 4, 5, 7].

The use of synthetic grafts such as Dacron and artificial ligaments, such as the Leeds-Keio ligament, allows the artificial ligament to bear most of the load in the early post-operative period. The advantage is that there is no donor-site morbidity. The disadvantages include an increased risk of infection and poor outcomes of revision TKA [8]. Ecker *et al*. [9] reported an excellent post-operative flexion angle of 146.4° when the Leeds-Keio ligament was used to repair damage to either the patella or quadriceps tendon in 11 patients, 1 of whom had bilateral patellar tendons repaired. However, artificial grafts and artificial ligaments have a wide tape-like structure that covers the patella’s surface, which means that the synthetic material extensively covers it. Therefore, infection from the superficial layer to the deeper layer is a concern, and there have been reports of infections occurring after reconstruction using artificial ligaments [10].

Autologous tissue grafts to augment the reconstruction of the extensor mechanism include semitendinosus and gracilis tendons, free fascia lata grafts, plantaris tendons and gastrocnemius flaps [1, 6, 10]. The semitendinosus and/or gracilis tendon is located medial to the patellar tendon and can be punctured through drill holes in the patella and then sutured to itself under appropriate tension [1, 6, 8, 11].

The advantage of this technique is that the patellar tendon can be securely fixed in a footprint and anatomically repaired without graft reinforcement, bone tunnels or prominent suture knots. Also, the disadvantage of this method is that the cost of repair using two anchors and flat-braided sutures outweighs the more convenient method using only sutures through a bone tunnel.

In the present cases, the patellar tendon rupture could have been caused by tendon fragility due to aging, associated microtrauma and increased extension during surgery. This repair method is unique, and the range of motion has been equivalent to the preoperative level until the final follow-up, with no extension delay, probably because of the absence of tissue loss or infection, and the optimum tension of the tendon could be restored easily. Thus, this repair method is unique in the sense that it is simple and easily reproducible, and encouraging results are expected in the current long-term follow-up.

Complications involving the extensor mechanism after TKA are disabling. Meticulous surgical techniques should be used in any patients undergoing TKA to prevent these complications from ever happening. Repair using a knotless anchor and flat-braided suture is a safe and effective method to treat patellar tendon rupture at the tuberosity despite the absence of any tissue loss.
